# Supplementary material for: A Bibliometric Visualization Analysis on Vaccine Development of Coronavirus Disease 2019 (COVID-19)
Source: Vaccines (Basel). 2023 Jan 29;11(2):295. doi: 10.3390/vaccines11020295 (PMC9959778; doi:10.3390/vaccines11020295)
Supplement: Supplementary file 1 [file vaccines-11-00295-s001.zip › Appendix S1 WHO approved 11 COVID-19 vaccines for the emergency use listing.pdf]

Supplementary Materials

**Appendix S1**	Table S1: WHO approved 11 COVID-19 vaccines for the emergency use listing (EUL)
**Appendix S2**	Table S2: Data from WOS core collection database download published in 2019_87<br>Articles n=87
**Appendix S3**	Table S3: Data from WOS core collection database download published in 2020_2400<br>Articles n=2400
**Appendix S4**	Table S4: Data from WOS core collection database download published in 2021_10290<br>Articles n=10290
**Appendix S5**	Table S5: Data from WOS core collection database download published in 2022_5508<br>Articles n=5508
**Appendix S6**	Table S6: Summary from web of science core collection database.
**Appendix S7**	Table S7: Publications on 11 WHO-approved COVID-19 vaccines for the emergency use listing
**Appendix S8**	Figure S1: The top-20 active journals and co-citation cited sources visualization map with in COVID-19 vaccine research by VOSviewer analysis
**Appendix S9**	Table S8: The retracted articles (n =10)

Table S1 WHO approved 11 COVID-19 vaccines for the emergency use listing (EUL)

Vaccine type	Trade name	WHO EUL Holder	Country	Number of doses	First recommendation issued
**messenger-RNA (mRNA) vaccine** COVD-19 mRNA Vaccine (nucleoside modified)	COMIRNATY®	BioNTech Manufacturing GmbH	Germany	6 doses of 0.3 mL (after dilution)	31 Dec 2020
**Recombinant adenovirus vector vaccine** COVID-19 Vaccine (ChAdOx1-S [recombinant])	VAXZEVRIA	AstraZeneca AB / SK Bioscience Co. Ltd AstraZeneca AB	Sweden	8 doses (0.5 mL per dose) and 10 doses	15 Feb 2021
**Recombinant adenovirus vector vaccine** COVID-19 Vaccine (ChAdOx1-S [recombinant])	COVISHIELD™	Serum Institute of India Pvt. Ltd	India	2 doses (0.5 mL per dose) and 10 doses	15 Feb 2021
**Recombinant adenovirus vector vaccine** COVID-19 Vaccine (Ad26.COV2-S [recombinant])	N/A	Janssen–Cilag International NV	Belgium	5 doses (0.5 mL per dose)	12 Mar 2021
**messenger-RNA (mRNA) vaccine**	SPIKEVAX	Moderna Biotech	Spain	10 doses (0.5	30 Apr 2021

COVID-19 mRNA Vaccine (nucleoside modified)		ModernaTX, Inc		mL per dose)	
**Inactivated vaccine**<br>Inactivated COVID-19 Vaccine (Vero Cell)	Not applicable	Beijing Institute of Biological Products Co., Ltd	China	1, 2 and 5 doses	07 May 2021
**Inactivated vaccine**<br>COVID-19 Vaccine (Vero Cell), Inactivated	CoronaVac	Sinovac Life Sciences Co., Ltd	China	1 dose and 2 doses of 0.5 mL each	01 Jun 2021
**Inactivated vaccine**<br>Covid-19 vaccine (Whole Virion Inactivated Corona Virus vaccine)	COVAXIN®	Bharat Biotech International Ltd	India	1, 5, 10 and 20 doses	03 Nov 2021
**Recombinant S proteins subunit vaccine**<br>COVID-19 vaccine (SARS-CoV-2 rS Protein Nanoparticle [Recombinant])	COVOVAX™	Serum Institute of India Pvt. Ltd	India	1 and 10 doses	17 Dec 2021
**Recombinant S proteins subunit vaccine**<br>COVID-19 vaccine (SARS-CoV-2 rS [Recombinant, adjuvanted])	NUVAXOVID™	Novavax CZ a.s.	Czech Republic	10 doses (0.5 mL per dose)	20 Dec 2021
**Recombinant adenovirus vector vaccine**	CONVIDECIA	CanSino Biologics Inc.	China	1 and 3 doses of	19 May 2022

COVID-19 Vaccine (Ad5-nCoV-S [Recombinant])				0.5 mL	
