# Supplementary material for: A Bibliometric Visualization Analysis on Vaccine Development of Coronavirus Disease 2019 (COVID-19)
Source: Vaccines (Basel). 2023 Jan 29;11(2):295. doi: 10.3390/vaccines11020295 (PMC9959778; doi:10.3390/vaccines11020295)
Supplement: Supplementary file 1 [file vaccines-11-00295-s001.zip › Appendix S7 Publications on 11 WHO-approved COVID-19 vaccines for the emergency use listing.pdf]

Supplementary Materials

**Appendix S1**	Table S1: WHO approved 11 COVID-19 vaccines for the emergency use listing (EUL)
**Appendix S2**	Table S2: Data from WOS core collection database download published in 2019_87<br>Articles n=87
**Appendix S3**	Table S3: Data from WOS core collection database download published in 2020_2400<br>Articles n=2400
**Appendix S4**	Table S4: Data from WOS core collection database download published in 2021_10290<br>Articles n=10290
**Appendix S5**	Table S5: Data from WOS core collection database download published in 2022_5508<br>Articles n=5508
**Appendix S6**	Table S6: Summary from web of science core collection database.
**Appendix S7**	Table S7: Publications on 11 WHO-approved COVID-19 vaccines for the emergency use listing
**Appendix S8**	Figure S1: The top-20 active journals and co-citation cited sources visualization map with in COVID-19 vaccine research by VOSviewer analysis
**Appendix S9**	Table S8: The retracted articles (n =10)

Table S7 Publications on 11 WHO-approved COVID-19 vaccines for the emergency use listing

Classification of vaccine	Vaccine type	Number	Trade name	Publications
**messenger-RNA (mRNA) vaccine**	COVD-19 mRNA Vaccine (nucleoside modified)<br>COVID-19 mRNA Vaccine (nucleoside modified)<br>COVID-19 Vaccine (Ad5-nCoV-S [Recombinant])	3	COMIRNATY®<br>SPIKEVAX<br>CONVIDECIA	1210 (63.2%)
**Recombinant adenovirus vector vaccine**	COVID-19 Vaccine (ChAdOx1-S [recombinant])<br>COVID-19 Vaccine (ChAdOx1-S [recombinant])<br>COVID-19 Vaccine (Ad26.COV2-S [recombinant])	3	VAXZEVRIA<br>COVISHIELD™<br>N/A	397 (20.7%)
**Inactivated vaccine**	Inactivated COVID-19 Vaccine (Vero Cell)<br>COVID-19 Vaccine (Vero Cell), Inactivated<br>Covid-19 vaccine (Whole Virion Inactivated Corona Virus vaccine)	3	Not applicable<br>CoronaVac<br>COVAXIN®	231(12.1%)
**Recombinant S proteins subunit vaccine**	COVID-19 vaccine (SARS-CoV-2 rS Protein Nanoparticle [Recombinant])<br>COVID-19 vaccine (SARS-CoV-2 rS [Recombinant, adjuvanted])	2	COVOVAX™<br>NUVAXOVID™	76(4.0%)
