# Supplementary material for: A Bibliometric Visualization Analysis on Vaccine Development of Coronavirus Disease 2019 (COVID-19)
Source: Vaccines (Basel). 2023 Jan 29;11(2):295. doi: 10.3390/vaccines11020295 (PMC9959778; doi:10.3390/vaccines11020295)
Supplement: Supplementary file 1 [file vaccines-11-00295-s001.zip › Figure S1. The top-20 active journals and co-citation cited sources.pdf]

Supplementary Materials

**Appendix S1**	Table S1: WHO approved 11 COVID-19 vaccines for the emergency use listing (EUL)
**Appendix S2**	Table S2: Data from WOS core collection database download published in 2019_87<br>Articles n=87
**Appendix S3**	Table S3: Data from WOS core collection database download published in 2020_2400<br>Articles n=2400
**Appendix S4**	Table S4: Data from WOS core collection database download published in 2021_10290<br>Articles n=10290
**Appendix S5**	Table S5: Data from WOS core collection database download published in 2022_5508<br>Articles n=5508
**Appendix S6**	Table S6: Summary from web of science core collection database.
**Appendix S7**	Table S7: Publications on 11 WHO-approved COVID-19 vaccines for the emergency use listing
**Appendix S8**	Figure S1: The top-20 active journals and co-citation cited sources visualization map with in COVID-19 vaccine research by VOSviewer analysis
**Appendix S9**	Table S8: The retracted articles (n =10)

Figure S1. The top-20 active journals and co-citation cited sources visualization map with in COVID-19 vaccine research by VOSviewer analysis.

(A) The enlarged map of Network visualization map of top cited sources (co-citations more than twenty-two)

(B) The density view of the journal map of top cited sources

(C) The enlarged map of top-20 highest TLS journals

The size of the nodes represented the citations frequency of journals, and journals with the same color in the view belong to the same clusters in cooperation network.

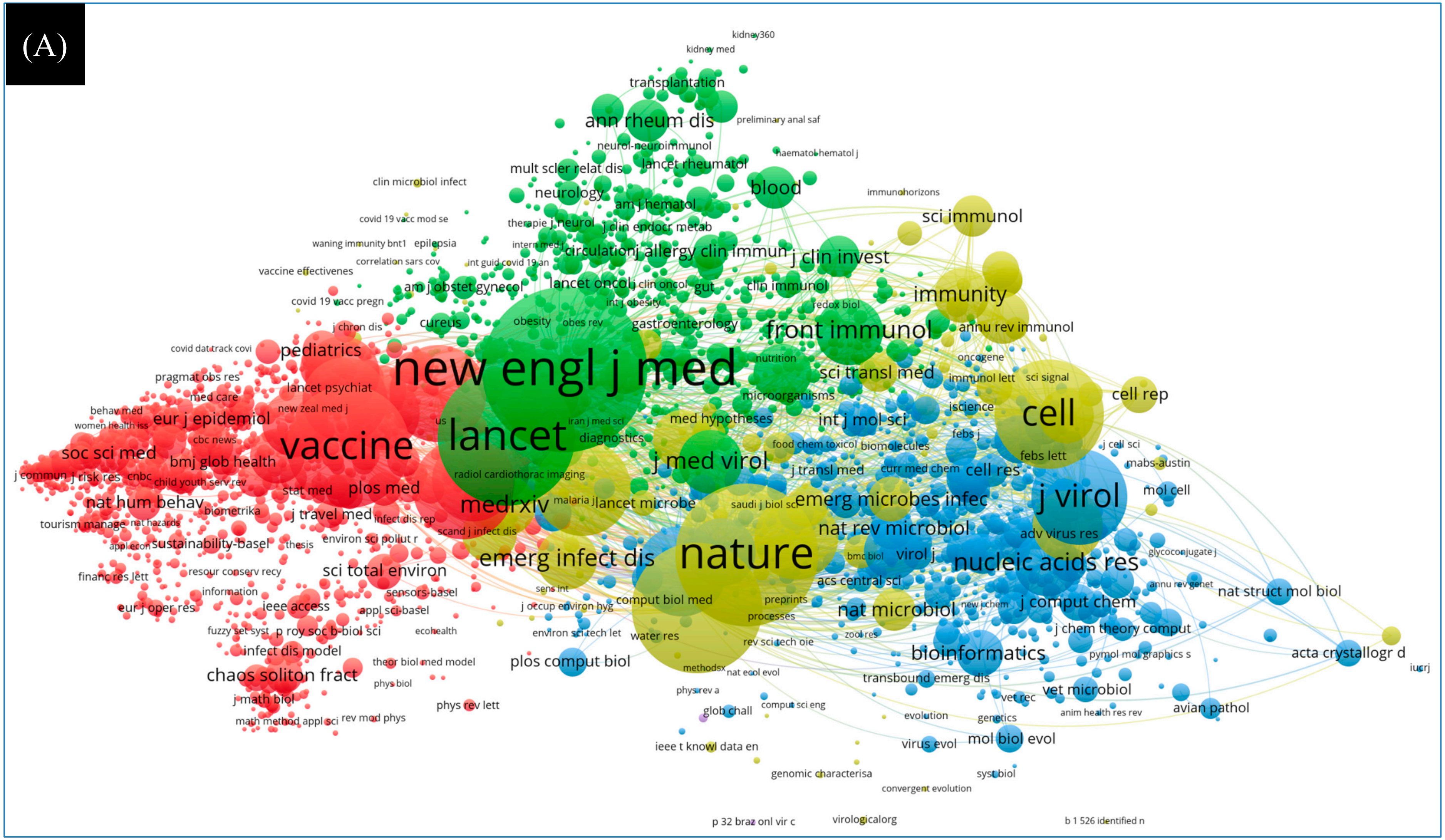
(A)
kidney360
kidney med
transplantation
ann rheum dis
preliminary anal saf
neurol-neuroimmunol
haematol hematol j
mult scler relat dis
lancet rheumatol
clin microbiol infect
neurology
blood
immunohorizons
am j hematol
covid 19 vacc mod se
therapie
j neurol
j clin endocr metab
sci immunol
waning immunity bnt1
epilepsia
correlation sars cov
int guid covid 19 an
circulation
j allergy clin immun
j clin invest
vaccine effectivenes
am j obstet gynecol
lancet oncol
j clin oncol
gut
clin immunol
immunity
covid 19 vacc pregn
int j obesity
redox biol
cureus
obesity
obes rev
gastroenterology
j chron dis
front immunol
annu rev immunol
covid dat track covi
pediatrics
nutrition
oncogene
new engl j med
sci transl med
immunol lett
sci signal
pragmat obs res
lancet psychiat
cell rep
med care
microorganisms
behav med
new zeal med j
iscience
women health iss
eur j epidemiol
iran j med sci
med hypotheses
int j mol sci
cell
lancet
febs j
diagnostics
j cell sci
cbc news
food chem toxicol
biomolecules
soc sci med
vaccine
febs lett
mabs-austin
j commun
j risk res
cnbc
bmj glob health
j med virol
child youth serv rev
radiol cardiothorac imaging
j transl med
curr med chem
cell res
stat med
plos med
mol cell
j virol
nat hum behav
medrxiv
malaria j
lancet microbe
saudi j biol sci
emerg microbes infec
biometrika
j travel med
infect dis rep
tourism manage
nat hazards
scand j infect dis
nat rev microbiol
adv virus res
environ sci pollut r
thesis
appl econ
sustainability-basel
emerg infect dis
nature
bmc biol
virol j
glycoconjugate j
nucleic acids res
financ res lett
resour conserv recy
sci total environ
acs central sci
annu rev genet
information
sens int
sensors-basel
comput biol med
preprints
nat struct mol biol
eur j oper res
ieee access
j occup environ hyg
nat microbiol
new j chem
j comput chem
appl sci-basel
processes
fuzzy set syst
p roy soc b-biol sci
ecohealth
environ sci tech let
water res
zool res
j chem theory comput
infect dis model
rev sci tech oie
bioinformatics
acta crystallogr d
pymol mol graphics s
theor biol med model
plos comput biol
iucrj
chaos soliton fract
methodsx
nat ecol evol
transbound emerg dis
phys biol
vet microbiol
phys rev a
vet rec
j math biol
phys rev lett
glob chall
comput sci eng
anim health res rev
avian pathol
math method appl sci
rev mod phys
evolution
genetics
ieee t knowl data en
virus evol
mol biol evol
genomic characterisa
syst biol
convergent evolution
p 32 braz onl vir c
virologicalorg
b 1 526 identified n

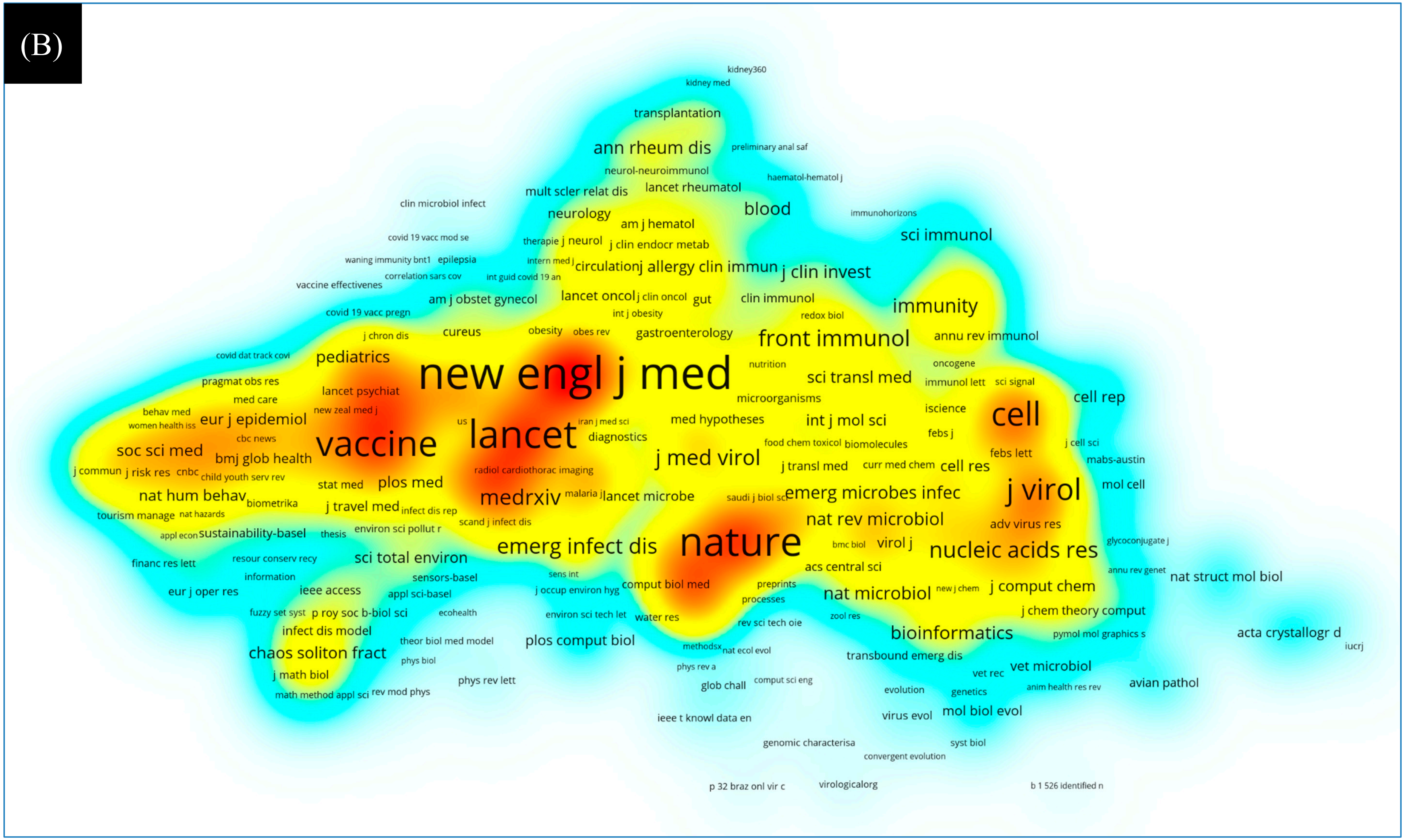
(B)
kidney360
kidney med
transplantation
ann rheum dis
preliminary anal saf
neurol-neuroimmunol
haematol-hematol j
mult scler relat dis
lancet rheumatol
clin microbiol infect
blood
immunohorizons
neurology
am j hematol
covid 19 vacc mod se
therapie
j neurol
j clin endocr metab
sci immunol
waning immunity bnt1
epilepsia
intern med j
circulation
j allergy clin immun
j clin invest
correlation sars cov
int guid covid 19 an
vaccine effectivenes
am j obstet gynecol
lancet oncol
j clin oncol
gut
clin immunol
immunity
covid 19 vacc pregn
int j obesity
redox biol
j chron dis
cureus
obesity
obes rev
gastroenterology
front immunol
annu rev immunol
covid dat track covi
pediatrics
new engl j med
nutrition
oncogene
pragmat obs res
lancet psychiat
sci transl med
immunol lett
sci signal
med care
microorganisms
cell rep
behav med
new zeal med j
iscience
women health iss
eur j epidemiol
us
lancet
iran j med sci
med hypotheses
int j mol sci
cell
cbc news
diagnostics
febs j
soc sci med
vaccine
food chem toxicol
biomolecules
j cell sci
bmj glob health
j med virol
febs lett
j commun
j risk res
cnbc
child youth serv rev
radiol cardiothorac imaging
j transl med
curr med chem
cell res
mabs-austin
stat med
plos med
j virol
mol cell
nat hum behav
biometrika
medrxiv
malaria j
lancet microbe
saudi j biol sci
emerg microbes infec
j travel med
infect dis rep
tourism manage
nat hazards
scand j infect dis
nat rev microbiol
adv virus res
appl econ
sustainability-basel
thesis
environ sci pollut r
glycoconjugate j
emerg infect dis
nature
bmc biol
virol j
nucleic acids res
financ res lett
resour conserv recy
sci total environ
acs central sci
annu rev genet
information
sensors-basel
sens int
nat struct mol biol
eur j oper res
ieee access
appl sci-basel
j occup environ hyg
comput biol med
preprints
nat microbiol
new j chem
j comput chem
processes
fuzzy set syst
p roy soc b-biol sci
ecohealth
environ sci tech let
water res
zool res
j chem theory comput
infect dis model
rev sci tech oie
bioinformatics
acta crystallogr d
theor biol med model
plos comput biol
methodsx
nat ecol evol
pymol mol graphics s
iucrj
chaos soliton fract
phys biol
transbound emerg dis
phys rev a
vet rec
vet microbiol
j math biol
phys rev lett
glob chall
comput sci eng
anim health res rev
avian pathol
math method appl sci
rev mod phys
evolution
genetics
ieee t knowl data en
virus evol
mol biol evol
genomic characterisa
syst biol
convergent evolution
p 32 braz onl vir c
virologicalorg
b 1 526 identified n

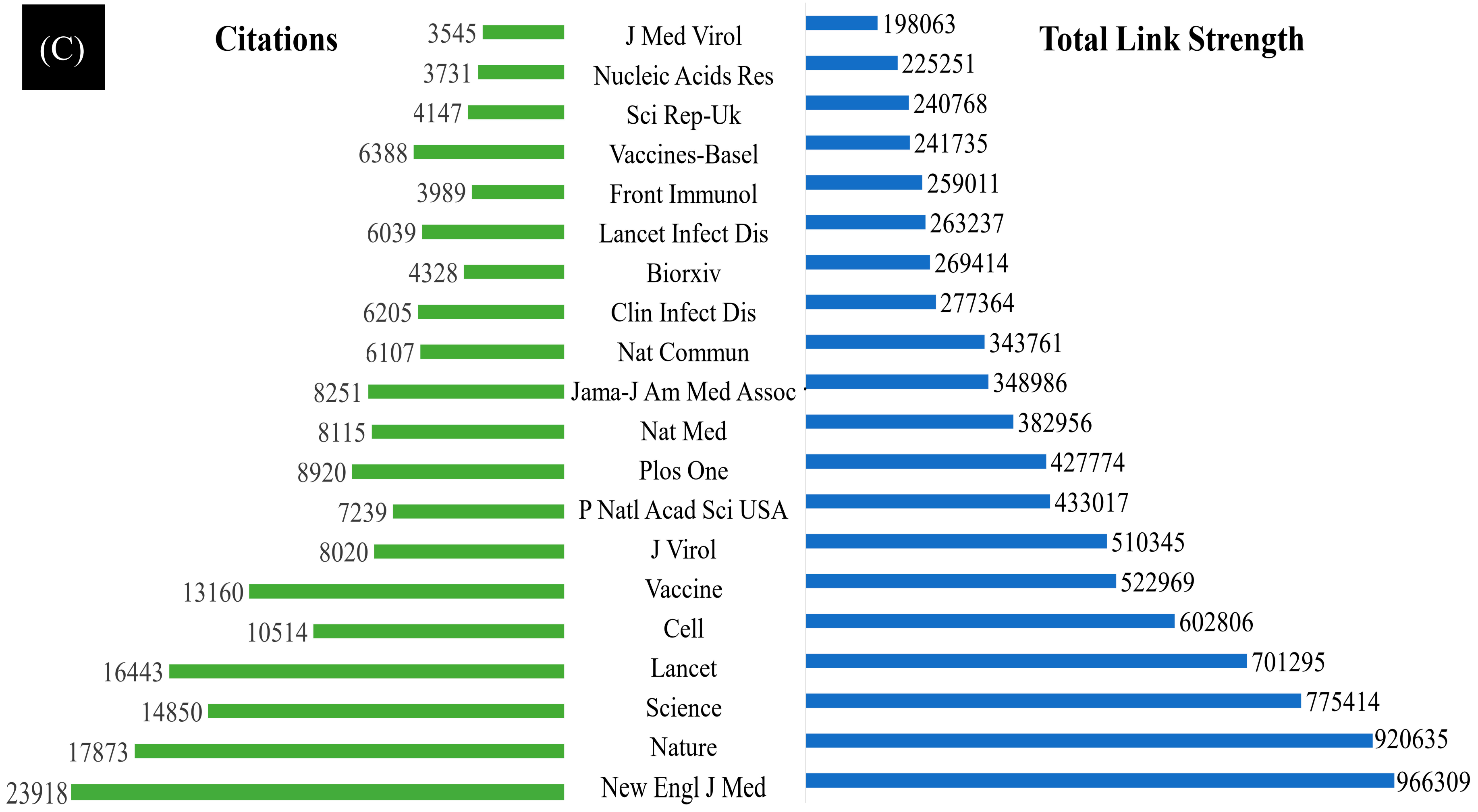
(C)
Citations
Total Link Strength
3545
J Med Virol
198063
3731
Nucleic Acids Res
225251
4147
Sci Rep-Uk
240768
6388
Vaccines-Basel
241735
3989
Front Immunol
259011
6039
Lancet Infect Dis
263237
4328
Biorxiv
269414
6205
Clin Infect Dis
277364
6107
Nat Commun
343761
8251
Jama-J Am Med Assoc
348986
8115
Nat Med
382956
8920
Plos One
427774
7239
P Natl Acad Sci USA
433017
8020
J Virol
510345
13160
Vaccine
522969
10514
Cell
602806
16443
Lancet
701295
14850
Science
775414
17873
Nature
920635
23918
New Engl J Med
966309
